# Temporal Trends in Suicide Attempts Among Children in the Decade Before and During the COVID-19 Pandemic in Paris, France

**DOI:** 10.1001/jamanetworkopen.2021.28611

**Published:** 2021-10-07

**Authors:** Anthony Cousien, Eric Acquaviva, Solen Kernéis, Yazdan Yazdanpanah, Richard Delorme

**Affiliations:** 1 Infection, Antimicrobials, Modeling, and Evolution Laboratory, French National Institute of Health and Medical Research, University of Paris, Paris, France; 2 Department of Child and Adolescent Psychiatry, University of Paris, Robert Debré University Hospital, Assistance Publique–Hôpitaux de Paris, Paris France; 3 Infection Prevention and Control Team, Bichat-Claude Bernard University Hospital, Assistance Publique–Hôpitaux de Paris, Paris, France; 4 Department of Infectious and Tropical Diseases, Bichat-Claude Bernard University Hospital, Assistance Publique–Hôpitaux de Paris, Paris, France

## Abstract

This cross-sectional study uses surveillance data to assess temporal trends in suicide attempts among children admitted to a pediatric emergency department in Paris, France, in the decade before and during the COVID-19 pandemic.

## Introduction

Recent studies have reported a deterioration in children’s mental health since the start of the COVID-19 pandemic in 2020, with an increase in anxiety and mood disorders.^1^ Rates of suicide ideation and suicide attempts among children were also higher when COVID-19–related stressors were heightened in 2020.^2,3^

We aimed to better assess temporal trends in suicide attempts among children while adjusting for annual and seasonal fluctuations. We conducted a cross-sectional study of surveillance data collected over the past 10 years at the Robert Debré Hospital in Paris, France, which is one of the largest pediatric centers in Europe.

## Methods

This cross-sectional study included all children aged 15 years or younger who attempted suicide and were admitted to the pediatric emergency department of Robert Debré Hospital between January 2010 and April 2021. The study comprised 830 children, with a mean (SD) age of 13.5 (1.5) years and a 1:4 ratio of boys to girls. A suicide attempt was defined as a nonfatal self-directed potentially injurious behavior with any intent to die as a result of the behavior.^4^ Deaths by suicide were not included in the analysis because those data were not available in real time.

For this study, we conducted a robust seasonal-trend decomposition using locally estimated scatterplot smoothing analysis on the bimestrial time series of all suicide attempts in the 2010 to 2021 surveillance data.^5^ The number of suicide attempts observed at time *t* was decomposed as (1) a seasonal component accounting for the variations observed during the year, (2) a long-term trend, and (3) a remainder. In the absence of atypical events, the remainder should be centered and uncorrelated and should have a constant variance.

This study followed the Strengthening the Reporting of Observational Studies in Epidemiology (STROBE) reporting guideline. The independent ethics committee of the Assistance Publique–Hôpitaux de Paris Health Data Warehouse approved the study protocol. Informed consent was not required, in accordance with French law regarding the retrospective use of monocentric anonymized routine data.

## Results

The deseasonalized time series results showed that the number of suicide attempts among children decreased from 12.2 at the lowest level (July to August) in 2019, to 7.8 during the first lockdown period (March to April) in 2020 in France (−36%; Figure; Table). However, the number of suicide attempts among children increased substantially from the lowest and highest levels of 12.2 (July to August) and 22.5 (November to December) in 2019, to 38.4 just before the second lockdown initiation (September and October) and 40.5 (early November to December) in 2020 (+116% and +299%, respectively). This aberrant dynamic of suicide attempts was independent from its annual seasonality and its trend over the 10-year period, as illustrated by the significant remainder components we observed after March 2020. The slope coefficient of remainders was different from 0 (*P* = .003), with a coefficient of −0.079 (95% CI, −0.13 to −0.027).

**Figure.**
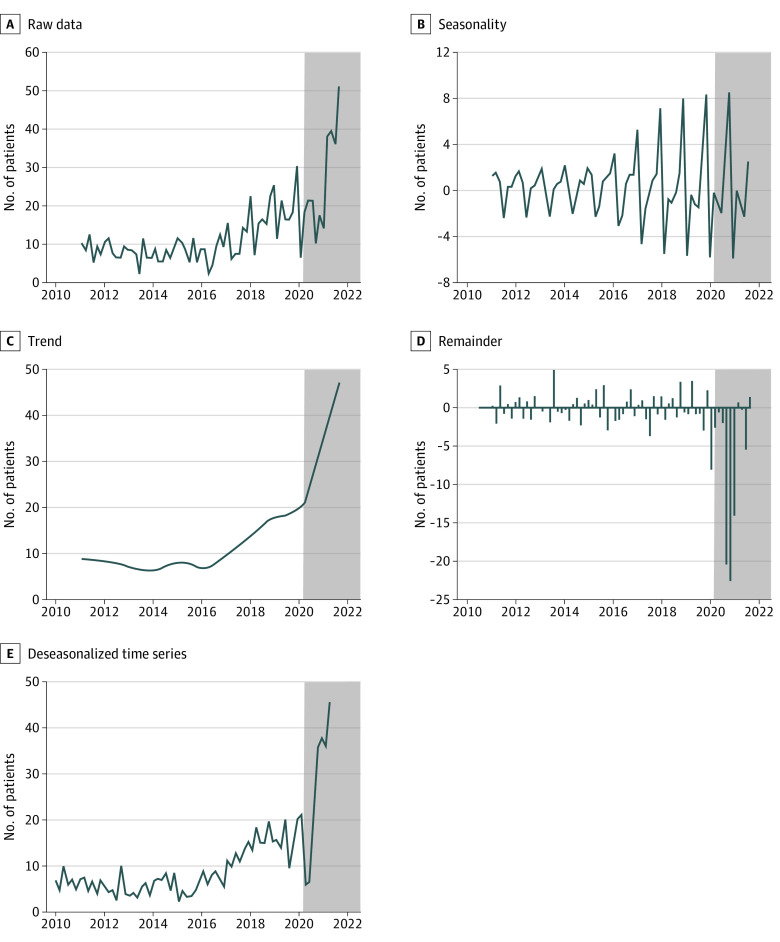
Robust Seasonal and Trend Decomposition Using LOESS Analysis^3^ of a Bimestrial Time Series of Suicide Attempts Among Children Younger Than 15 Years in the Decade Before and During the COVID-19 Pandemic in Paris, France These surveillance data are for 830 children admitted to the pediatric emergency department of Robert Debré Hospital in Paris, France, between January 2010 and April 2021. Shaded data from March 2020 onward indicate the period after the World Health Organization declared the COVID-19 outbreak a pandemic. A, Raw data on the number of suicide attempts during 10 years of follow-up. B, Estimation of suicide attempt seasonality. We observed an annual seasonality effect on the occurrence of suicide attempts among children. C, Long-term trend of the series. We observed a progressive increase in suicide attempts during the last 10 years (January 2010 and April 2021) in France, as also reported in the US.^4^ D, Based on our model, the STL analysis revealed atypical events in the series of suicide attempts since the pandemic onset. Outliers in the remainder corresponded to anomalies in the time series (ie, signals not explained by the seasonality and long-term trend). E, The deseasonalized time series represented the sum of the trend and the remainder components. We observed a decrease in suicide attempts during the first lockdown period in France (March to April 2020) but a major increase (in September and October 2020) just before the initiation of the second lockdown (in early November 2020). LOESS indicates locally estimated scatterplot smoothing; STL, seasonal and trend decomposition using LOESS.

**Table.** Deseasonalized Adjusted Numbers of Suicide Attempts Among Children Aged Younger Than 15 Years in France, by Bimesters From 2019 to 2021

Year	Bimester					
	Jan/Feb	Mar/Apr	May/Jun	Jul/Aug	Sep/Oct	Nov/Dec
2019	17.8	16.1	21.8	12.2	18.5	22.5
2020	23.3	7.8	8.6	20.3	38.4	40.5
2021	38.6	48.7	NA	NA	NA	NA

NA, not available.

## Discussion

Since 2010, the incidence of suicide attempts among children has increased worldwide.^4^ Our analysis further suggests a dramatic increase in suicide attempts among children in late 2020 and early 2021 after the start of the COVID-19 pandemic in France.

Similar results in the dynamics of suicide, suicide attempts, or suicide ideations among children have been reported recently.^2,3,6^ Many factors may have contributed to this acceleration, such as children’s specific sensitivity to mitigation measures, deterioration of family health and economic conditions, increased screen time and social media dependence, or bereavement. Interestingly, we also observed a decrease in suicide attempts in the early months of the pandemic during the March 2020 lockdown period in France, which may have resulted from not only increased parental supervision but also difficulties in accessing urgent care.

Our study had some limitations in that it was monocentric, subject to representativeness bias, and also underpowered to stratify patients by sex. Only patients aged 15 years or younger were included, which limits the comparison of our findings with similar works that include patients aged 16 to 21 years. Finally, our careful monitoring of potential changes in children’s mental health since the pandemic onset may also have led to an overestimation of the outcomes we reported.

In conclusion, our findings suggest that the COVID-19 pandemic is associated with profound changes in the dynamics of suicide attempts among children. There is a need for rapid deployment of evidence-based prevention and intervention strategies to address factors influencing suicide attempts among children during and likely after the pandemic.
